# Low‐grade serous carcinoma detected from intraoperative peritoneal washings: Cytological findings and detection of KRAS mutation

**DOI:** 10.1002/cnr2.1676

**Published:** 2022-07-08

**Authors:** Eriko Yamamoto, Kenji Warigaya, Yuichi Kinoshita, Ayana Yamamoto, Shin‐ichi Murata

**Affiliations:** ^1^ Division of Diagnostic Pathology, Department of Central Laboratory Medicine Wakayama Medical University Hospital Wakayama Wakayama Japan; ^2^ Division of Diagnostic Pathology Shin‐kuki General Hospital Kuki Saitama Japan; ^3^ Department of Human Pathology Wakayama Medical University Wakayama Wakayama Japan

**Keywords:** cytological findings, KRAS mutation, low‐grade serous carcinoma (LGSC), ovary, peritoneal washings

## Abstract

**Background:**

Low‐grade serous carcinoma (LGSC) of the ovary, which is extremely rare tumor, has better prognosis than high‐grade serous carcinoma (HGSC). Genetic backgrounds of those are different, so that LGSC usually shows KRAS or BRAF mutation, whereas HGSC does not show such mutations. Since treatment strategies of those are different, differential pathological diagnosis between LGSC and HGSC is very important.

**Case:**

We report a case of LGSC that was diagnosed by both cytological findings and genetic analysis using small amount cells from cytological specimen. The 30‐year‐old Japanese woman with bilateral ovarian tumors underwent salpingo‐oopherectomy. The peritoneal washing cytologic specimen and touched cytologic specimen from the tumor included non‐complex clusters with psammoma bodies composed of tumor cells with mild to moderate atypia and without bizarre nuclei. The ovarian tumor was histologically diagnosed as LGSC. The genetic analysis that was done using exfoliated cells from peritoneal washings specimen by idensy™, detected KRAS mutation at codon 12/13.

**Conclusion:**

The genetic investigation using cytological specimen as well as characteristic cytological findings were useful to make differential diagnosis between LGSC and HGSC.

## 1 INTRODUCTION

Ovarian serous carcinomas account for 46%–67% of all ovarian carcinomas.^1^ Ovarian serous carcinoma can be differentiated into the more common high‐grade ovarian serous carcinoma (HGSC) and the very rare low‐grade serous carcinoma (LGSC) variant, which has been described as comprising fewer than 5% of all cases.^1^, ^2^ Almost all LGSCs and HGSCs are detected incidentally or following the onset of nonspecific symptoms and present at an advanced stage frequently occurring with bilateral ovarian masses.^2^ HGSCs occur frequently in patients aged 55–65 years, grow rapidly, and respond well to chemotherapy, with a 5‐year survival rate of 9%–34%.^3^ However, LGSCs occur frequently in younger patients (mean age, 45–57 years), grow slowly, and demonstrate a poor response to chemotherapy with a 5‐year survival rate of 40%–56%.^3^ Additionally, some reports have described distinct differences in the genetic mutations underlying these tumors, with HGSCs usually displaying a high level of chromosomal instability and *TP53* mutations, and LGSCs usually demonstrating mutations in *KRAS* or *BRAF*.^2^, ^3^, ^4^ Here, we describe the cytological characteristics of an extremely rare case of LGSC from an intraoperative peritoneal washing specimen determined using immunohistochemical and genetic analyses.

## 2 CASE PRESENTATION

A 30‐year‐old Japanese woman with type 2 diabetes mellitus and without reproductive or birth history has a familial history of breast cancer but no familial history of ovarian cancer. The patient was admitted to our hospital because of lower abdominal pain. A pelvic ultrasound revealed a cystic and solid mass with a diameter of approximately 120 mm in her right ovary. Total hysterectomy with bilateral salpingo‐oophorectomy and omentectomy was performed. The patient was followed up without any sign of recurrence for 2 years after surgery.

### 2.1 Cytological findings

**FIGURE 1:**
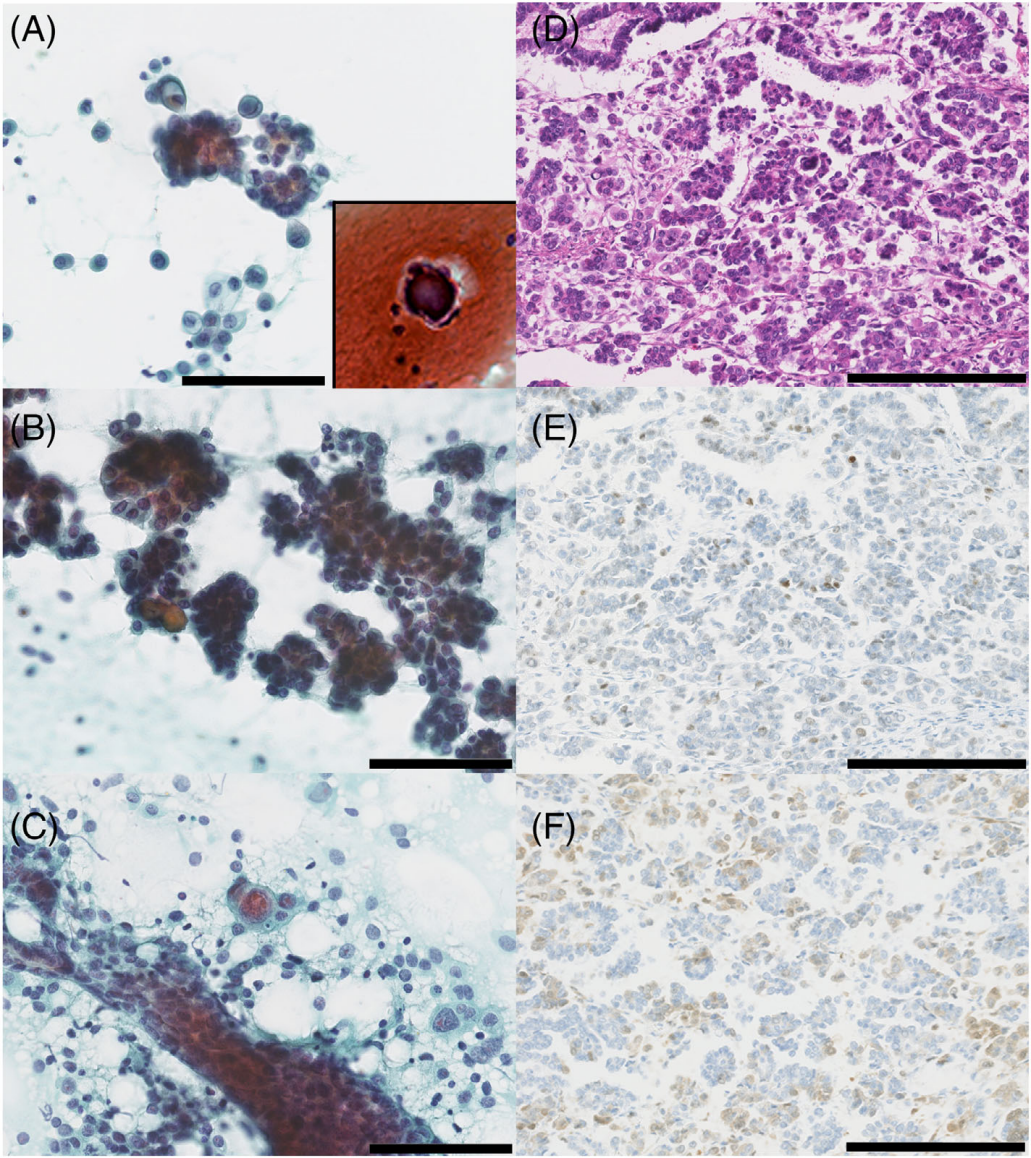
Representative morphological findings. Cytological findings for low‐grade serous carcinoma (LGSC) in the peritoneal washing (A) and touched (B) cytological specimens from the tumor (B), visualized using Papanicolaou staining (original magnification, ×400). Flat, sheet‐like, papillary and micropapillary cell clusters were observed. Neoplastic cells show mild‐to‐moderate atypia with a high nuclear‐to‐cytoplasmic ratio, peripherally located swollen nuclei, irregular nuclear contours, and increased fine granular chromatin. Psammoma bodies are also seen (inset in A). Typical cytological findings of a high‐grade serous carcinoma (HGSC) case are shown (C) for comparison (Papanicolaou stain, original magnification, ×400). HGSC cells showed moderate‐to‐marked atypia with scattered pleomorphic nuclei. There should be no pleomorphic cells when trying to distinguish between LGSC and HGSC (A–C). Representative histological findings for the LGSC invasive lesion, visualized using H&E stain (D) (original magnification, ×200). Immunohistochemical staining for p53 revealed that these samples were characterized by scattered, weakly positive neoplastic cells (E) and p16 is weakly positive for approximately 40% of tumor cells (F) (original magnification, ×200) (scale bar; A–C: 100 μm, D–F: 250 μm)

Representative cytological findings for the peritoneal washing (Figure 1A) and touched tumor specimens (Figure 1B) are shown. We found many flat, sheet‐like, and micropapillary clusters of atypical glandular cells. Neoplastic cells displayed a high nuclear‐to‐cytoplasmic content ratio, peripherally located nuclei, three‐dimensional irregular nuclear contours, and increased fine chromatin. Additionally, psammoma bodies were occasionally observed.^2^ Moreover, when compared with control HGSC samples (Figure 1C), the neoplastic cells had uniform medium‐sized nuclei; they also did not display pleomorphic nuclei, which were scattered across in the HGSC tissues.

### 2.2 Histological and immunohistochemical findings

The surgically resected right ovary contained a cystic lesion with a yellowish‐white solid component. Histological examination revealed both papillary and micropapillary structures with invasive lesions in the solid component (Figure 1D). Psammoma bodies were found to be scattered across these samples and neoplastic epithelial cells presented with a high nuclear‐to‐cytoplasmic content ratio, uniform medium‐sized nuclei, coarse nuclear chromatin, and conspicuous nucleoli. Immunohistochemical evaluation revealed that approximately 20% of the neoplastic cells were weakly positive for p53 (Figure 1E), whereas 40% of these cells were weakly positive for p16 (Figure 1F). The surgically resected left ovary had a cystic lesion classified as a serous borderline tumor (SBT). We also found disseminated tumors in the right pelvic peritoneum, left fallopian tube, and omentum. Based on the histological, immunohistochemical, and genetic findings, we settled on a diagnosis of ovarian LGSC.

### 2.3 Genetic investigation

**FIGURE 2:**
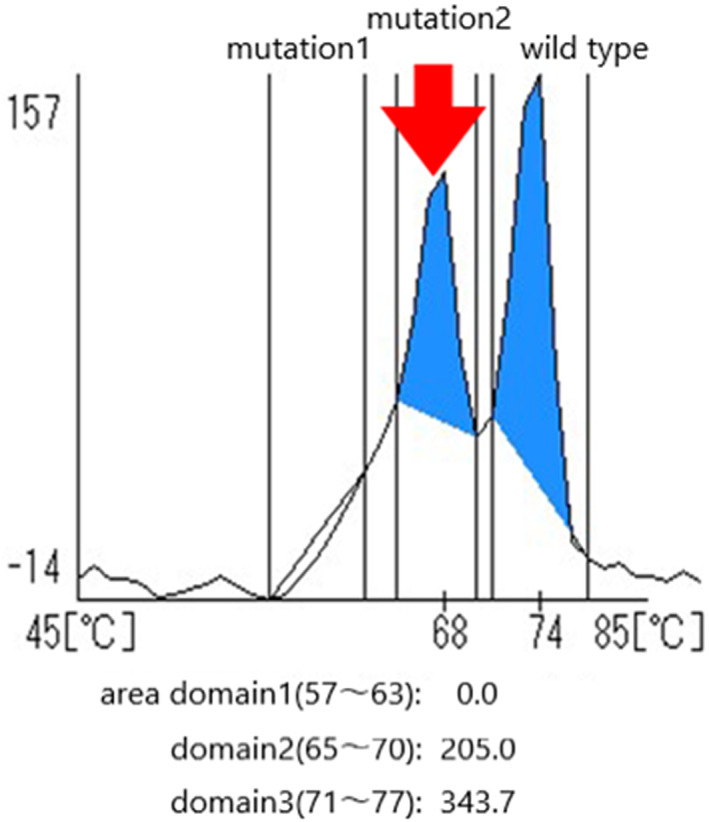
Results of the *KRAS* gene evaluations analyzed using i‐densy™ IS‐5320. The tumor cell DNA was extracted from the tumor cells derived from the intraoperative peritoneal washing specimen. The histogram indicates that these cells include a *KRAS* codon 12/13 mutation

To investigate *KRAS* and *BRAF* genetic mutations, we extracted DNA from tumor cells obtained from the peritoneal washing cytological specimen. We identified a *KRAS* mutation at codon 12/13, but no *BRAF* mutation using an i‐densy™ IS‐5320 (Arkray, Kyoto) based on quenching probe method (Figure 2).^5^ We confirmed the same genetic mutation at both LGSC cells of the right ovary and SBT cells of the left ovary, which were obtained from the surgical histological sections.

## 3 DISCUSSION

Both LGSC and HGSC are invasive carcinomas of the ovary; however, their response to chemotherapy and prognosis are markedly different. Because HGSC is highly sensitive to chemotherapy, chemotherapy based on platinum preparation is usually performed after the primary debulking surgery, and neoadjuvant chemotherapy can be administered in cases of bulky tumors.^6^ In contrast, LGSC is resistant to chemotherapy, making surgery the first‐line treatment for LGSC.^7^ Therefore, distinguishing LGSC from HGSC via cytology prior to surgery and determining their intraoperative cytology is important to ensure proper therapeutic management and prevent unnecessary chemotherapy.

Histologically, LGSC demonstrates invasive growth with small nested, tubular, papillary, micropapillary, and inverted micropapillary structures with mildly to moderately atypical cells. Solid and macropapillary structures, frequently found in HGSC, are uncommon in LGSC.^3^ Mitotic activity is also significantly lower in these tumors, and necrosis is almost never found. However, both HGSC and LGSC display scattered psammoma bodies.^3^

A few previous reports have briefly described cytological findings in LGSC, but none have provided detailed descriptions.^8^, ^9^, ^10^ Our cytological examination of LGSC revealed isolated atypical epithelial cells and variable cellular clusters of sheet‐like, tubular, papillary, and micropapillary structures. LGSC cells are usually small‐to‐medium‐sized and have a high nuclear‐to‐cytoplasmic content ratio, nuclei with small irregularities, increased coarse chromatin, and prominent nucleoli.^8^, ^9^ The psammoma bodies from these LGSC cell clusters are useful for the differential diagnosis of non‐serous carcinomas. These cytological features are very similar to those of SBT; therefore, they cannot be used to distinguish between LGSC and SBT.^8^, ^9^, ^11^ HGSCs usually have more complicated three‐dimensional structures, such as branching papillary and cribriform patterns, which are composed of pleomorphic tumor cells with various nuclear‐to‐cytoplasmic ratios, intracytoplasmic vacuoles, and marked nuclear atypia. Despite the differences in their cytological findings, most cytopathologists still struggle with a differential cytological diagnosis of HGSC and LGSC when, the patient presents with moderate cellular atypia like this case. We believe that the bizarre nuclei, which are detected in HGSC, but not in LGSC, are one of the most important features to differentially diagnose LGSC and HGSC.

Carcinogenesis of LGSC is still unclear, even though there were some studies showing its relation with diabetes mellitus like the present case. There are differences of genetic abnormalities between LGSC and HGSC. Unlike HGSC, LGSC is characterized by a lack of abnormal p53 expression^4^ and block‐positive p16 expression. Additionally, 50%–60% of LGSC tumors have *KRAS* or *BRAF* mutations, whereas HGSC is not associated with documented mutations in either gene.^2^, ^3^ Here, we successfully detected *KRAS* mutations in a small number of tumor cells taken from the intraoperative peritoneal washing cytological samples collected during resection. These successful results indicate that cytological specimens can be used for genetic evaluation, improving cytology‐based differential diagnosis.^11^ In conclusion, our cytological findings suggest that non‐complex clusters with psammoma bodies composed of tumor cells with mild‐to‐moderate atypia and without bizarre nuclei are clear cytological indicators of LGSC. In addition, our findings indicate the usefulness of investigating KRAS mutations using DNA extracted from cytological specimens to aid in the differential diagnosis of LGSC and HGSC.

## AUTHOR CONTRIBUTIONS

Eriko Yamamoto and Kenji Warigaya: Diagnosis of the case and preparation of this manuscript. Yuichi Kinoshita and Ayana Yamamoto: Analysis of immunohistochemical stain and KRAS mutation. Sin‐ichi Murata: Editing and reviewing of this manuscript. *Writing – Original Draft*, E.Y., K.W.; *Investigation*, Y.K., A.Y.; *Writing – Review & Editing*, S‐I, M.

## CONFLICT OF INTEREST

The authors have stated explicitly that there are no conflicts of interest in connection with this article.

## ETHICS STATEMENT

This case report does not meet the requirements for institutional approval or patient consent and is exempt.

## PATIENT CONSENT STATEMENT

Patient consent has been obtained and is available if requested.

## Data Availability

Data are available on request from the authors.
